# Lifetime Residential Mobility History and Self-Rated Health at Midlife

**DOI:** 10.2188/jea.JE20110055

**Published:** 2012-03-05

**Authors:** Kuan-Chia Lin, Hui-Chuan Huang, Ya-Mei Bai, Pei-Chun Kuo

**Affiliations:** 1 School of Nursing, National Taipei University of Nursing and Health Sciences, Taiwan; 2 Life-Course Epidemiology and Human Development Research Group, National Taipei University of Nursing and Health Sciences, Taiwan; 3 Department of Psychiatry, Taipei Veterans General Hospital, Taipei, Taiwan; 4 Department of Psychiatry, Faculty of Medicine, National Yang-Ming University, Taipei, Taiwan; 5 Department of Nursing, Cardinal Tien College of Healthcare & Management, Taiwan

**Keywords:** residential mobility, self-rated health, personal context, residential environmental satisfaction

## Abstract

**Background:**

Little research focuses on the influence of lifetime residential mobility on health at midlife. We used a national survey of participant recall of residential mobility to assess this issue and explore the mediating and moderating effects of personal and environmental context.

**Methods:**

In March 2010, we collected data from people in Taiwan aged 40 to 60 years. Based on the household registration system, data were collected using the population proportional-to-size sampling method and a computer-assisted telephone interview. A total of 2834 participants completed the interview. Based on the 3490 registered households, the overall response rate was 81.2%.

**Results:**

The mean cumulative frequency of geographic relocation (CFGR) was 3.06 ± 2.78 times and ranged from 0 to 21. After carefully adjusting for the heterogeneity of demographic and socioeconomic propensity, total CFGR was significantly positively associated with negative self-rated mental (odds ratio [OR] and 95% CI for increase per time: 1.06, 1.02–1.16) and physical (OR and 95% CI for increase per time: 1.16, 1.05–1.26) health. Social network support lessened the impact of total CFGR on self-rated mental health. In addition to the primary effect, the interaction (residential environmental satisfaction × total CFGR) significantly moderated negative mental health and negative physical health.

**Conclusions:**

Lifetime residential mobility history independently influenced midlife health. Social network support and satisfaction with the residential environment in past and current living places further mediated or moderated midlife health. Findings from these different perspectives offer insights for future medical care projects and epidemiologic studies.

## INTRODUCTION

Residential mobility during a person’s life involves multiple interactions between this individual and the social environment and can result in a lasting impact.^1^^,^^2^ As compared with other events in a person’s life, residential mobility is unique as it is not entirely negative and can be related to numerous other contextual factors, such as social network support and environmental issues.^3^^,^^4^ Demographers have noted that the long- and short-term psychological and physical effects of moving are related to interactions between the above contextual factors and the socioeconomic characteristics of the individual before and after geographic relocation.^5^

Several prior studies have shown that early childhood experiences of mobility are related to academic performance, risky behaviors, and personality development, which might be due to the influence of frequent relocation on factors such as the stability of family structure, constancy of environment, development of interpersonal relationships, and access to health services.^6^^–^^10^ Many experts believe that geographic relocation is closely correlated with the accumulation of social network support within the framework of personal development.^11^^,^^12^ In addition to childhood, geographic relocation can also be a major life event in adolescence and adulthood.^13^^,^^14^ Young and middle-aged adults frequently cite the desire to maximize their income or job satisfaction through educational attainment or new employment opportunities as reasons for geographic relocation.

Life-course epidemiology focuses on the development of risk and elaboration of the interpretation of the health-environment association.^1^^,^^15^^,^^16^ As such, environmental measurements are crucial to life-course studies because an individual’s environment might moderate, or even mediate, the effects of biological risks.^17^^–^^20^ Frequent geographic relocation in conjunction with different spatial characteristics, neighborhood disorders, and associated conditions can also have interactive effects on people’s psychosocial conditions. More specifically, several theoretical models have emphasized the importance of post-relocation adaption to a new environment.^3^^,^^4^^,^^17^ For example, mobility experience theory and the hypothesis regarding history of previous relocation have shown that mobility is not simply an event with specific outcomes, but a set of social and environmental experiences that, when combined, result in successful or unsuccessful adaption to a new environment.^3^^,^^17^ These theorists argue that the effects of residential mobility can be moderated by the history of previous migrations or post-relocation adaption^4^^,^^17^ and that it is the “fit” between the person and the residential environment that is important.^18^

Numerous population studies have investigated the developmental impact of early mobility experiences on young adolescents; however, few studies have investigated possible changes in health at midlife due to moving during different stages of life. On the basis of previous research, we hypothesize that the overall impact of residential instability gradually limits one’s life-course, depending on the number and/or duration of exposures. Over many years, Taiwan has undergone major changes in social environment, and it and other societies have become increasingly mobile. According to publications by the Directorate-General of Budget, Accounting and Statistics, Taiwan, the mobility rate for this decade is between 6% and 10%, and the annual number of migrants to Taiwan ranges from 1.4 to 2.3 million.^21^

Therefore, this research used national surveys of participant residential mobility experiences to investigate the impact of life-course residential mobility history on midlife health and assess the mediating and moderating roles of personal and environmental context on middle-aged people who have experienced frequent geographic relocation.

## METHODS

### Study population

Data for this study were collected from March through May 2010. The sampling framework for people aged 40 to 60 years was obtained from the household registration system of Taiwan. To obtain a representative sample, we used a 2-phase randomized sampling procedure. In phase 1, the sample size was estimated based on an assumed prevalence (P ≈ 10% to 15%) of mobility, which was based on the results of earlier surveys in Taiwan.^21^ Additional parameters considered in estimating sample size were type I error (α = 0.05) and type II error (β = 0.2), and we determined that a sample size of at least 400 subjects was required from each sampling unit.^22^ Because Taiwan has 25 administrative divisions for the household registration system, this study divided the 25 administrative divisions into 7 sampling areas (north, north-central, central, south-central, south, northeast, and east), which provided a total sample size of 2800 (400 × 7 = 2800). During the next phase, subjects from each administrative division of the various sampling areas were selected using the population proportional-to-size method, which is designed to yield self-weighting and gives every eligible subject an equal chance of being selected. Finally, 2834 research participants completed the interview. The overall response rate was 81.2% based on the target representative surveys of 3490 by the household registration. Ethical approval was obtained from the institutional review board of the Department of Nursing, National Taipei College of Nursing (ID: 98A211).

### Outcome variable and information on geographic relocation

*We* developed the questionnaire based on a literature review and in consultation with experts on geographic relocation. The questionnaire, which was assessed using content validity, was peer reviewed by 5 experts (3 specialists in epidemiology/public health with doctoral degrees and 1 expert each in demography and sociology) to assess the correlation between the objective of the study and the content and questions. The dependent variables were 2 dimensions of subjective health status: self-rated mental and physical health. The single-item, global measure of self-rated health has been reported to be a useful tool for charting health status and a good proxy evaluation of overall health changes in adults.^23^^,^^24^ Therefore, respondents were asked to rate their present mental and physical health as either very poor, poor, fair, good, or excellent. Negative mental and physical health scores were defined as a response of very poor or poor. The primary independent variable of interest in the analyses was cumulative frequency of geographic relocation (CFGR).^6^ For the convenience of participants, we adopted a strict definition of geographic relocation. During the interview process, the question was as follows: “We would like to know the conditions of residential mobility since childhood.” By this we meant a residence longer than 6 months before and after moving, excluding international migration. To assist recall of residential mobility, participants were questioned about relocations during different stages of their lives. The stages were: before age 7, between 7 and 12 (elementary school), between 13 and 17, from 18 to the present, and within the last 5 years (eg, “Do you remember how many times you relocated between 13 and 17 years of age, while you were in junior high school and senior high school, and lived for more than 6 months before and after moving?”). The content validity of individual items on the scales (I-CVI) was rated (range 0.84–1.00), which is the minimum acceptable criteria corresponding to adequate content validity. A computer-assisted telephone interview (CATI) was then conducted to collect data.^25^ CATI technology permitted us to combine standardized call-back procedures, computer-controlled skip patterns, and data cleaning into a single operation, thereby bypassing the traditional paper-and-pencil coding and data entry procedures. All interviews were conducted by trained interviewers who could speak the major ethnic languages.

### Other study parameters

The support provided by social networks was defined using 2 measures: positive relationships with friends and positive relationships with extended family.^12^ The questions were: “How much does your family truly care about you and understand the way you feel?” and “How much can you rely on your friends for help if you have a serious problem or if you need to talk about your concerns?”. Scores ranged from 1 (not at all) to 4 (a lot). The notion of satisfaction with residential environment in this study was defined as the attitude of a resident (or household) towards the living environment.^26^ Measures of the level of satisfaction with residential environment included: (a) How satisfied are you with your overall past living place? (b) How satisfied are you with your present living place? Scores ranged from 1 (very dissatisfied) to 4 (very satisfied) and were coded as poor (for very dissatisfied/dissatisfied) and good (for satisfied/very satisfied). To compare levels of residential environmental satisfaction in past and current living places, the responses to the above 2 measures were then cross-classified as from poor to poor, from good to poor, from poor to good, and from good to good.

Demographic factors (age, sex, marital status, and employment), socioeconomic status (level of educational attainment and household income), and other background characteristics were assessed to evaluate potential conflicting effects. For employment, occupation was categorized as: (1) workers in agriculture, forestry, fishing, or animal husbandry, (2) unemployed individuals not seeking work, (3) unemployed individuals seeking work, (4) retired/disabled, (5) technicians and related occupations, (6) non-technicians and laborers, (7) administrative staff or service workers, (8) unpaid family workers, and (9) professionals.

### Analytic approach and statistical analysis

The Figure (adapted from Kuh et al) shows the schematic framework for the different patterns of risk exploration used in this study.^15^^,^^16^ First, CFGR during various life stages is hypothesized to have independent effects on the mental and physical health of middle-aged adults. Second, a clustering of risks is further examined, and the impact of total CFGR is hypothesized to be mediated or moderated by later exposures, such as various personal characteristics, level of social support, and residential environmental satisfaction. Data are summarized as the mean ± standard deviation for continuous variables and as proportions for categorical variables. Regarding variation, the median CFGR and interquartile range (IQR) are presented. Interrelationships of midlife health with CFGR and the characteristics of the study population were examined by using the *t* test with unequal variance and the chi-square test. From this point, total CFGR was used to illustrate the overall relationship, and the 3 CFGR categories included in the categorical comparisons were used to make detailed inferences regarding the life-course effects of residential mobility. Multiple logistic regression was used to analyze the contribution of personal context and satisfaction with the environment to the relationship between history of residential mobility and midlife health. The analysis and modeling strategies comprised 3 steps. In adjusted model 1, CFGR was treated as the major independent variable and was adjusted for demographics and socioeconomic status for possible confounding effects. Due to the degree of interrelationships among the various demographic characteristics, we used propensity scores for the adjustment.^27^ This method uses a weighted analysis that directly compares the means marginalized over the confounders to yield unbiased estimates of the residential parameters. Each respondent is assigned a weight (W_i_) equal to the inverse of the propensity score for residential mobility (X_i_) according to their demographic characteristics (Z_i_), for example, W_i_ = 1/Pr[X_i_ = x_i_|Z_i_ = z_i_]. In steps 2 and 3 (adjusted models 2 and 3), the independent effect of total CFGR was further challenged by adding the level of social network support and satisfaction with residential environment. In these steps, the possible interactions of total CFGR with social network support and satisfaction with residential environment were also examined. The above statistical analyses were conducted using SPSS version 18 (SPSS, Inc., Chicago, IL, USA).

**Figure.**
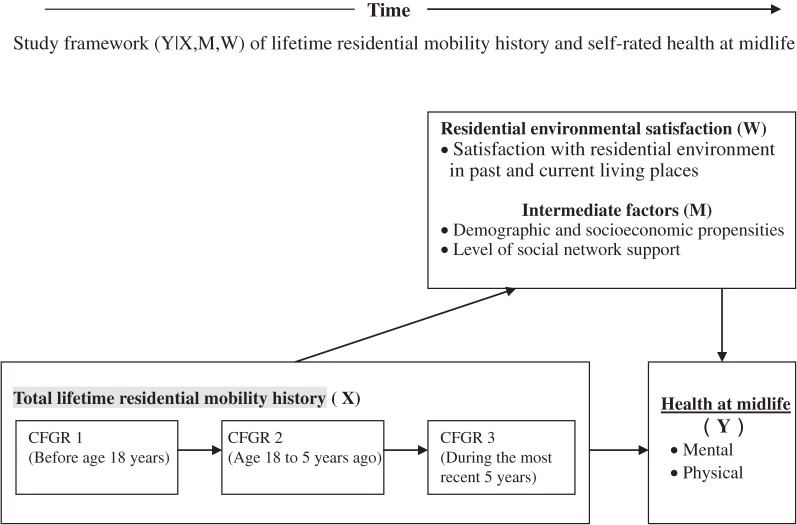
Schematic of possible mediating and moderating roles of personal and environmental context for residential mobility history and self-rated health; boxes indicate variables, and arrows indicate the hypothesized causal, mediating, or moderating effects. CFGR: cumulative frequency of geographic relocation.

## RESULTS

This study enrolled 2834 respondents: 1409 men and 1425 women. The mean frequency of CFGR was 3.06 ± 2.78 times, the median was 2 (IQR 3), and the range was 0 to 21. Table 1 shows the associations between relocation frequency during the life stages of the participants and the study variables, ie, demographics, socioeconomic status, social support, and environmental satisfaction. Univariate analyses indicated that the mobility histories of middle-aged adults were heterogeneous with regard to demographic and socioeconomic propensity. Additionally, the results in Table 1 show that the level of self-perceived social network support and environmental satisfaction in past and current living places were significantly associated with mobility history.

**Table 1.** Interrelationships between cumulative frequency of geographic relocation (CFGR) and the demographic characteristics, socioeconomic status, social support, and residential environmental satisfaction of the study participants

Parameters	*n* (%)	Total CFGR		CFGR (Before age 18 years)				CFGR (Age 18 to 5 years ago)				CFGR (During the most recent 5 years)		
			
		*M* ± SD	*P*	0 (%)	1–2 (%)	≥3 (%)	*P*	0 (%)	1–2 (%)	≥3 (%)	*P*	0 (%)	≥1 (%)	*P*
**Sex**														
Male	1409 (49.7%)	2.84 ± 3.05	0.148	52.1	32.8	15.0	0.129	30.6	43.3	26.1	<0.001	88.8	11.2	0.057
Female	1425 (50.3%)	3.01 ± 2.48		56.0	30.0	14.0		8.3	64.3	27.4		91.0	9.0	
**Age, yrs**														
40–44	707 (25.0%)	2.93 ± 2.72	0.008	43.8	37.9	18.3	<0.001	24.7	55.0	19.7	<0.001	89.3	10.7	0.001
45–49	723 (25.0%)	2.62 ± 2.51		55.1	32.9	11.9		21.4	54.5	24.1		86.6	13.4	
50–54	700 (24.7%)	2.97 ± 2.87		57.6	27.8	14.7		19.0	51.1	29.8		91.9	8.1	
55–60	705 (24.8%)	3.12 ± 2.86		61.0	26.0	13.0		12.2	54.6	33.2		92.5	7.5	
**Education level**														
Low	1099 (38.8%)	2.73 ± 2.77	<0.001	65.1	22.0	12.9	<0.001	20.6	50.7	28.8	<0.001	91.8	8.2	0.001
Medium	941 (33.2%)	2.70 ± 2.54		52.5	34.1	13.4		21.3	57.4	21.2		90.4	9.6	
High	794 (28.0%)	3.44 ± 3.01		41.4	40.8	18.0		15.7	54.0	30.3		86.8	13.2	
**Marital status**														
Married	2447 (87.1%)	2.87 ± 2.68	<0.001	55.3	30.8	13.8	<0.001	17.3	56.5	26.2	<0.001	90.9	9.4	0.006
Unmarried	168 (6.0%)	2.52 ± 2.76		40.9	43.5	15.6		46.4	34.3	19.3		86.2	13.8	
Separated	87 (3.1%)	4.83 ± 4.54		46.3	26.3	27.5		25.6	24.4	50.0		80.5	19.5	
Other	109 (3.9%)	3.46 ± 2.57		49.5	30.1	20.4		13.2	50.0	36.8		89.0	11.0	
**Household income (NTD)**														
0–20 000	257 (10.2%)	2.76 ± 2.96	0.028	62.5	21.3	16.3	<0.001	31.0	45.2	23.8	<0.001	86.7	13.3	0.007
20 000–60 000	1310 (52.2%)	2.88 ± 2.89		55.3	30.0	14.7		18.8	56.0	25.3		91.6	8.4	
60 000–100 000	595 (23.7%)	3.00 ± 2.63		48.1	35.9	16.0		17.0	55.2	27.8		87.2	12.8	
≥100 000	346 (13.8%)	3.37 ± 2.72		46.4	39.8	13.8		14.4	49.6	36.0		88.2	11.8	
**Employment**														
Agriculture/Forestry/Fishing/Animal husbandry	102 (3.6%)	1.23 ± 1.89	<0.001	78.0	17.0	5.0	<0.001	52.5	40.4	7.1	<0.001	96.1	3.9	<0.001
Unemployed individuals not searching for work	70 (2.5%)	2.96 ± 3.76		58.5	24.6	16.9		29.0	42.0	29.0		95.7	4.3	
Unemployed individuals searching for work	106 (3.8%)	2.90 ± 3.05		53.5	28.3	18.2		30.1	40.8	29.1		94.3	5.7	
Retired/disabled	139 (4.9%)	3.52 ± 3.05		52.0	24.0	24.0		16.8	48.9	34.3		87.8	12.2	
Technicians and related occupations	445 (15.8%)	2.88 ± 2.33		47.3	35.9	16.8		18.7	57.3	24.0		85.1	14.9	
Non-technicians and laborers	244 (8.7%)	2.45 ± 2.20		61.9	28.0	10.2		22.3	55.4	22.3		92.2	7.8	
Administrative staff or service workers	392 (13.9%)	2.86 ± 2.45		55.6	33.2	11.2		25.3	45.0	29.7		91.8	8.2	
Unpaid family workers	638 (22.7%)	2.82 ± 2.91		61.5	25.4	13.1		6.9	66.1	27.0		92.1	7.9	
Professionals	678 (24.1%)	3.35 ± 3.17		44.6	38.6	16.8		20.0	51.1	28.9		87.6	12.4	

**Social network support**														
Positive relations with extended family														
Good	2443 (90.0)	2.79 ± 2.56	<0.001	55.4	31.4	13.2	<0.001	19.4	54.8	25.8	0.037	90.3	9.7	0.089
Poor	270 (10.0)	3.92 ± 4.12		42.3	33.8	23.8		18.7	48.3	33.0		87.0	13.0	
Positive relations with friends														
Good	2462 (89.9)	2.87 ± 2.67	0.005	54.5	31.5	14.0	0.050	19.2	54.3	26.5	0.421	89.8	10.2	0.823
Poor	277 (10.1)	3.37 ± 3.60		48.5	32.4	19.1		20.9	50.2	28.9		90.3	9.7	
**Satisfaction with residential environment in past and current living places**														
Poor → Poor	136 (4.8)	3.65 ± 2.76	<0.001	36.7	43.0	19.4	0.021	14.0	49.5	36.6	<0.001	87.2	12.8	0.018
Good → Poor	198 (7.0)	3.27 ± 3.20		45.8	33.8	20.4		17.6	61.5	20.9		87.1	12.9	
Poor → Good	432 (15.2)	3.83 ± 2.81		45.9	34.4	19.7		7.0	56.7	36.4		84.9	15.1	
Good → Good	2068 (73.0)	3.07 ± 2.67		50.9	34.7	14.4		11.1	60.9	28.0		90.2	9.8	

NTD, new Taiwan dollars (1 US dollar ≈ 31 NTD).

Table 2 shows the interrelationships of midlife health with cumulative CFGR and the characteristics of the study population. The total CFGR for participants with poor self-rated mental health was 3.63 ± 3.49, as compared with 2.85 ± 2.68 among subjects with good self-rated mental health. Similarly, the total CFGR was 3.66 ± 3.47 among subjects with poor self-rated physical health and 2.84 ± 2.65 among subjects with good self-rated physical health. The results indicate that a higher CFGR during any life stage (particularly before age 18 and within the last 5 years) was associated with a higher rate of poor midlife health. Approximately 18% of participants who had moved more than 3 times before age 18 had poor self-rated health. During the period from age 18 to 5 years ago, around 15% of participants who had moved more than 3 times had poor self-rated health. However, at this stage, respondents with a CFGR of 1 to 2 had the highest proportion of good self-rated health. Additionally, regarding moves during the most recent 5 years, we found that around 15% of participants who had moved at least once had poor self-rated health. However, the rate of poor midlife health was also significantly positively correlated with educational level, marital status, household income, employment profile, poor social network support, and worsening satisfaction with residential environment (ie, transition from “poor to poor” or “good to poor” between past and current living places).

**Table 2.** Interrelationships of midlife health with cumulative frequency of geographic relocation (CFGR) and the demographic characteristics, socioeconomic status, social support, and residential environmental satisfaction of the study participants

Parameters			Self-rated mental health			Self-rated physical health		
	
			*Poor* *M* ± SD	*Good* *M* ± SD	*P*	*Poor* *M* ± SD	*Good* *M* ± SD	*P*
**Total CFGR**			3.63 ± 3.49	2.85 ± 2.68	0.003	3.66 ± 3.47	2.84 ± 2.65	0.002

		*n* (%)	*Poor*, %	*Good*, %	*P*	*Poor*, %	*Good*, %	*P*

**CFGR** (Before age 18 years)								
	**0**	1533 (51.4%)	8.8	91.2	0.012	11.1	88.9	0.001
	**1–2**	890 (31.4%)	10.1	89.9		12.4	87.6	
	**≥3**	391 (14.5%)	17.2	82.8		18.3	81.7	
**CFGR** (Age 18 to 5 years ago)								
	**0**	549 (19.4%)	12.7	87.3	0.021	12.2	87.8	0.552
	**1–2**	1528 (53.9%)	9.2	90.8		12.4	87.6	
	**≥3**	757 (26.7%)	15.3	84.7		13.9	86.1	
**CFGR** (During the most recent 5 years)								
	**0**	2457 (89.9%)	10.3	89.7	0.022	10.8	89.2	0.025
	**≥1**	285 (10.1%)	15.8	84.2		14.8	85.2	
**Sex**	Male	1409 (49.7%)	11.7	88.3	0.132	11.9	88.1	0.130
	Female	1425 (50.3%)	9.9	90.1		13.8	86.2	
**Age, yrs**	40–44	707 (25.0%)	10.9	89.1	0.102	11.0	89.0	0.043
	45–49	723 (25.0%)	8.6	91.4		11.5	88.5	
	50–54	700 (24.7%)	12.7	87.3		15.6	84.4	
	55–60	704 (24.8%)	10.8	89.2		13.7	86.3	
**Education level**	Low	1099 (38.8%)	13.8	86.2	<0.001	16.7	83.3	<0.001
	Medium	941 (33.2%)	9.5	90.5		12.2	87.8	
	High	794 (28.0%)	8.1	91.9		8.4	91.6	
**Marital status**	Married	2447 (87.1%)	9.4	90.6	<0.001	12.0	88	0.003
	Unmarried	168 (6.0%)	15.0	85		17.5	82.5	
	Separated	87 (3.1%)	33.3	66.7		22.6	77.4	
	Others	109 (3.9%)	17.9	82.1		17.0	83.0	
**Household income (NTD)**								
	0–20 000	257 (10.2%)	24.8	75.2	<0.001	24.8	75.2	<0.001
	20 000–60 000	1310 (52.2%)	11.7	88.3		13.9	86.1	
	60 000–100 000	595 (23.7%)	5.3	94.7		8.5	91.5	
	≥100 000	346 (13.8%)	6.6	93.4		8.7	91.3	
**Employment**								
Agriculture/Forestry/Fishing/Animal husbandry		102 (3.6%)	10.8	89.2	<0.001	14.1	85.9	<0.001
Unemployed individuals not searching for work		70 (2.5%)	15.2	84.8		29.9	70.1	
Unemployed individuals searching for work		106 (3.8%)	19.8	80.2		22.5	77.5	
Retired/disabled		139 (4.9%)	13.3	86.7		17.2	82.8	
Technicians and related occupations		445 (15.8%)	6.6	93.4		10.3	89.7	
Non-technicians and laborers		244 (8.7%)	11.5	88.5		11.6	88.4	
Administrative staff or service workers		392 (13.9%)	11.7	88.3		16.8	83.2	
Unpaid family workers		638 (22.7%)	15.4	84.6		11.1	88.9	
Professionals		678 (24.1%)	7.4	82.6		8.3	91.7	

**Social network support**								
Positive relations with extended family								
	Good	2443 (90.0)	8.0	92.0	<0.001	11.1	88.9	<0.001
	Poor	270 (10.0)	31.3	68.7		26.0	74.0	
Positive relations with friends								
	Good	2462 (89.9)	8.2	91.8	<0.001	11.3	88.7	<0.001
	Poor	277 (10.1)	28.9	71.1		25.0	75.0	
**Satisfaction with residential environment** **in past and current living places**								
	Poor → Poor	136 (4.8)	36.6	63.4	<0.001	23.4	76.6	<0.001
	Good → Poor	198 (7.0)	38.2	61.8		28.9	71.1	
	Poor → Good	432 (15.2)	10.6	89.4		18.4	81.6	
	Good → Good	2068 (73.0)	6.4	93.6		9.7	90.3	

NTD, new Taiwan dollars (1 US dollar ≈ 31 NTD).

Tables 3 and 4 show the results of multiple logistic regression analysis of self-rated negative mental and physical health at midlife, respectively. First, the results revealed that a higher CFGR before age 18 was significantly associated with negative self-rated mental and physical health. CFGR during the most recent 5 years was also significantly positively associated with self-rated mental and physical heath. In addition, after adjusting the propensity score, total mobility history remained significantly associated with negative self-rated mental and physical health. In the adjusted Model 3, the effects of social network support lessened the impact of total CFGR on self-rated mental health, but the effect on self-rated physical health was limited. In the adjusted Model 3, the impact of satisfaction with residential environment was further increased and examined. The results showed that worsening environmental satisfaction (from good to poor) was the most significant risk factor for poor midlife health. In addition to the main effect, 2 significant interactions were found after the interaction terms (residential environmental satisfaction × total CFGR) were introduced at this step.

**Table 3.** Odds ratio (ORs) and 95% confidence interval (CI) in statistical modeling to test the effects of cumulative frequency of geographic relocation (CFGR) with self-rated mental health

Frequency		Negative self-rated mental health		

		OR^b^ [95% CI]	OR^c^ [95% CI]	OR^d^ [95% CI]
**Model A**^a^ **Residential mobility (categorical comparisons)**				
CFGR 1 (Before age 18 years)	**≥3 vs. 0**	2.03 [1.10–2.70]	1.72 [1.52–2.31]	1.70 [1.52–2.35]
	**1–2 vs. 0**	1.22 [0.82–1.64]	1.13 [0.84–1.52]	1.13 [0.79–1.59]
CFGR 2 (Age 18 to 5 years ago)	**≥3 vs. 0**	1.25 [0.92–1.76]	1.23 [0.88–1.79]	1.23 [0.84–1.85]
	**1–2 vs. 0**	0.71 [0.43–1.07]	0.71 [0.42–1.07]	0.75 [0.44–1.17]
CFGR 3 (During the most recent 5 years)	**≥1 vs. 0**	1.83 [1.32–2.73]	1.82 [1.28–2.76]	1.73 [1.22–2.67]

**Model B** **Residential mobility (increase per time)**				
Total CFGR		1.20 [1.10–1.25]	1.07 [1.02–1.15]	1.06 [1.02–1.16]
**Add social support**				
Negative relations with extended family (y/n)			2.96 [2.04–4.31]	2.31 [1.37–3.24]
Negative relations with friends (y/n)			3.11 [2.08–4.64]	2.71 [1.77–4.14]
**Add satisfaction with residential environment in past and current living places**				
RES1: Poor → Poor vs. Good → Good				2.89 [1.15–6.46]
RES2: Good → Poor vs. Good → Good				6.26 [3.98–9.83]
RES3: Poor → Good vs. Good → Good				1.31 [0.86–1.98]
RES1 × Total mobility				1.34 [1.06–1.70]
RES2 × Total mobility				1.56 [0.92–3.05]
RES3 × Total mobility				0.86 [0.73–0.95]

^a^Only final models are presented for inferences regarding the 3 CFGR categories (categorical comparisons).

^b^Adjusted model 1: adjusted for demographic characteristics by propensity score analysis.

^c^Adjusted model 2: adjusted for social network support.

^d^Adjusted model 3: adjusted for residential environmental satisfaction.

**Table 4.** Odds ratio (ORs) and 95% confidence interval (CI) in statistical modeling to test the relationships of cumulative frequency of geographic relocation (CFGR) with self-rated physical health

Frequency		Negative self-rated physical health		

		OR^b^ [95% CI]	OR^c^ [95% CI]	OR^d^ [95% CI]
**Model A**^a^ **Residential mobility (categorical comparisons)**				
CFGR 1 (Before age 18 years)	**≥3 vs. 0**	1.97 [1.42–2.72]	1.93 [1.37–2.69]	1.90 [1.34–2.64]
	**1–2 vs. 0**	1.14 [0.87–1.55]	1.12 [0.83–1.49]	1.12 [0.83–1.49]
CFGR 2 (Age 18 to 5 years ago)	**≥3 vs. 0**	1.32 [0.86–2.10]	1.30 [0.83–2.06]	1.29 [0.82–2.04]
	**1–2 vs. 0**	1.10 [0.73–1.66]	1.12 [0.74–1.72]	1.12 [0.73–1.72]
CFGR 3 (During the most recent 5 years)	**≥1 vs. 0**	1.48 [1.16–1.87]	1.40 [1.09–1.78]	1.33 [1.04–1.69]

**Model B** **Residential mobility (increase per time)**				
Total CFGR		1.16 [1.08–1.23]	1.16 [1.07–1.25]	1.16 [1.05–1.26]
**Add social support**				
Negative relations with extended family (y/n)			1.88 [1.30–2.73]	1.54 [1.04–2.33]
Negative relations with friends (y/n)			1.95 [1.35–2.81]	1.82 [1.22–2.75]
**Add satisfaction with residential environment in past and current living places**				
RES1: Poor → Poor vs. Good → Good			1.85 [1.25–2.57]	2.89 [1.15–6.46]
RES2: Good → Poor vs. Good → Good			4.62 [2.59–8.23]	6.26 [3.98–9.83]
RES3: Poor → Good vs. Good → Good			1.49 [0.76–2.18]	1.31 [0.86–1.98]
RES1 × Total mobility			1.24 [1.06–1.69]	1.34 [1.06–1.70]
RES2 × Total mobility			1.49 [0.88–2.97]	1.56 [0.92–3.05]
RES3 × Total mobility			0.87 [0.71–0.94]	0.86 [0.73–0.95]

^a^Only final models are presented for inferences regarding the 3 CFGR categories (categorical comparisons).

^b^Adjusted model 1: adjusted for demographic characteristics by propensity score analysis.

^c^Adjusted model 2: adjusted for social network support.

^d^Adjusted model 3: adjusted for residential environmental satisfaction.

These significant interactions indicated that the effect of residential mobility history on midlife health was moderated by the level of environmental satisfaction regarding the past and current living places. More specifically, the results showed significant synergistic effects between worsening environmental satisfaction (from poor to poor) and total mobility on both negative mental and physical health (OR = 1.34 for mental health and OR = 1.24 for physical health). In contrast, significant antagonistic effects between improved environmental satisfaction (from poor to good) and total mobility on both negative mental and physical health (OR = 0.86 for mental health and OR = 0.87 for physical health) were observed.

## DISCUSSION

After carefully adjusting for the heterogeneity of demographic and socioeconomic propensity, this study revealed an independent association between mobility history and self-rated health at midlife. Social support was a partial mediator that helped explain the persistent effects of mobility history on mental health. We also found that variation in satisfaction with residential environment in past and current living places significantly moderated the interrelationships between residential mobility history and self-rated health.

Previous studies indicated that unstable living environments are associated with socioeconomic factors such as housing, unemployment, change in family structure, and single parenting, which, together with relocation, are significant coexisting determinants of health status.^2^^,^^5^^–^^7^^,^^12^ Our study adds to the current literature and confirms that life-course residential mobility history, especially migration experiences before age 18 and during the most recent 5 years, have a long- or short-term effect on midlife self-rated health. It has been suggested that frequent moving is an indicator of greater family problems and can cause emotional stress during early life.^9^^,^^10^^,^^12^^,^^28^^,^^29^ Previous studies of youth have shown increasingly negative effects with more relocation experiences.^13^^,^^14^ In addition, with regard to life-course transitions, mobility experiences from adolescence to adulthood might further reflect an individual’s long-term residential stability context. This may subsequently interact at the levels of neighborhood, family, and individual in cumulative and compounding ways (development of social skills and health behaviors, access to health care, and sense of control over one’s environment), with significant effects on adult health.^6^^–^^8^^,^^12^^,^^13^^,^^17^ Because social context during various life stages can affect health trajectories during life, the impact of frequent geographic relocation was hypothesized to begin in childhood and accumulate over one’s life.^6^^,^^12^^,^^13^ The findings of the present study support the hypothesis that more frequent geographic relocation throughout life adversely influences self-rated health and that this influence persists into midlife.

Nevertheless, we found no significant relationship when migration experience after age 18 to 5 years ago was included in the study model. Moreover, subjects with a moderate level of relocations during adulthood had the highest proportion of good self-rated health. These findings suggest that frequent relocation does not increase the risk of poor health in a straightforward manner. Higher CFGR among individuals with higher socioeconomic status responded (Table 2) may partially explain these associations. Therefore, the strength and direction of health effects due to relocation might vary by socioeconomic status and could be important factors in the late-life relocation process in the longitudinal trajectory of health.

The results showed that social support partially lessened the impact of total CFGR and migration experience before age 18 on self-rated mental health at midlife. Lu and colleagues proposed that the impact, and mediating pathways, of family separation due to residential mobility tend to have a immediate and detrimental effect on psychological and physical health.^30^ This disruption to family life probably reduces the size and level of social support, which diminishes the emotional well-being of migrants. Chinese people have a tradition of fostering an extended family, and immediate relatives tend to live together or within a short distance; therefore, participants who experienced a higher frequency of relocation in early life may have experienced more variability in connections and more volatile relationships with relatives. Residential stability was also proven to increase people’s connection to social and institutional networks, thereby providing them the opportunity to develop strong social and community ties.^13^^,^^14^^,^^31^^–^^34^

This study investigated the moderating role of environmental satisfaction on the relationship between residential mobility history and midlife health. The synergistic effects of worsening environmental satisfaction suggest that as post-relocation environmental satisfaction declines, the negative effects of frequent residential mobility, and its influence on the health of middle-aged adults, increase. Previous theories and empirical research have indicated that relocations caused by factors perceived as negative increase the difficulty of adjustment, while post-relocation adjustment is easier in relocations precipitated by factors perceived as positive (moving for a better job or for better housing and environment).^3^^,^^4^^,^^17^^,^^35^^–^^38^ In contrast, the antagonistic effects of improved environmental satisfaction also suggest that when people have positive satisfaction with their residential environment, frequent geographic relocations may not be so harmful. Therefore, an increase in the probability of poor health in a moderated environment is either due to a decrease in satisfaction with the destination, relatively higher satisfaction with the previous location, or both.^39^^–^^41^ These findings suggest that policies which focus only on the immediate relationship between residential mobility and health miss a significant moderator: change in satisfaction with the residential environment. Additionally, migrants who experience declining environmental satisfaction might require mental and physical health care. Further research on the social indicators (population density, median income, and crime rate) of declining environmental satisfaction is required to identify the moderating influence of location on the relationship between geographic relocation and mental/physical health.

Several limitations of this study, and promising directions for future research, warrant mention. Regarding the representative sample, the research participants recalled their residential mobility history; thus, recall bias was possible. However, we used a strict definition of mobility—residence in locations for longer than 6 months before and after relocation—which should have limited recall bias to significant mobility experiences. Additionally, relocation is a complex transition, and we have examined only a limited array of the participants’ socioeconomic characteristics at midlife. An important component of adaptation that was not included in this analysis is change in socioeconomic status, which is associated with residential mobility and might have an important effect on self-rated health.^42^ A more complete picture of changes in socioeconomic status during each life stage of relocation will emerge when such factors are integrated into the explanatory model, which will require further study. Moreover, the interview process also limited our ability to obtain more specific information on each life stage, such as medical care, the trajectories of positive and negative life events, and the development of psychological characteristics. Future research might produce explicit causal models by examining time-varying factors and the effects of various constructs of health status on lifetime residential mobility history. Finally, the study results were applied specifically to a midlife Chinese population; therefore, studies of other ethnic populations are necessary.

In summary, our findings suggest that lifetime residential mobility history is independently associated with midlife health. Social network support and satisfaction with the residential environment in past and current living places further mediate and moderate midlife health. From the different perspectives provided, the findings of this study offer insight for future projects on medical care and epidemiologic studies.
